# Supplementary material for: mTORC2 Is the Major Second Layer Kinase Negatively Regulating FOXO3 Activity
Source: Molecules. 2022 Aug 24;27(17):5414. doi: 10.3390/molecules27175414 (PMC9457944; doi:10.3390/molecules27175414)
Supplement: Supplementary file 1 [file molecules-27-05414-s001.zip › molecules-1850492-supplementary.pdf]

Supplementary Figure S1

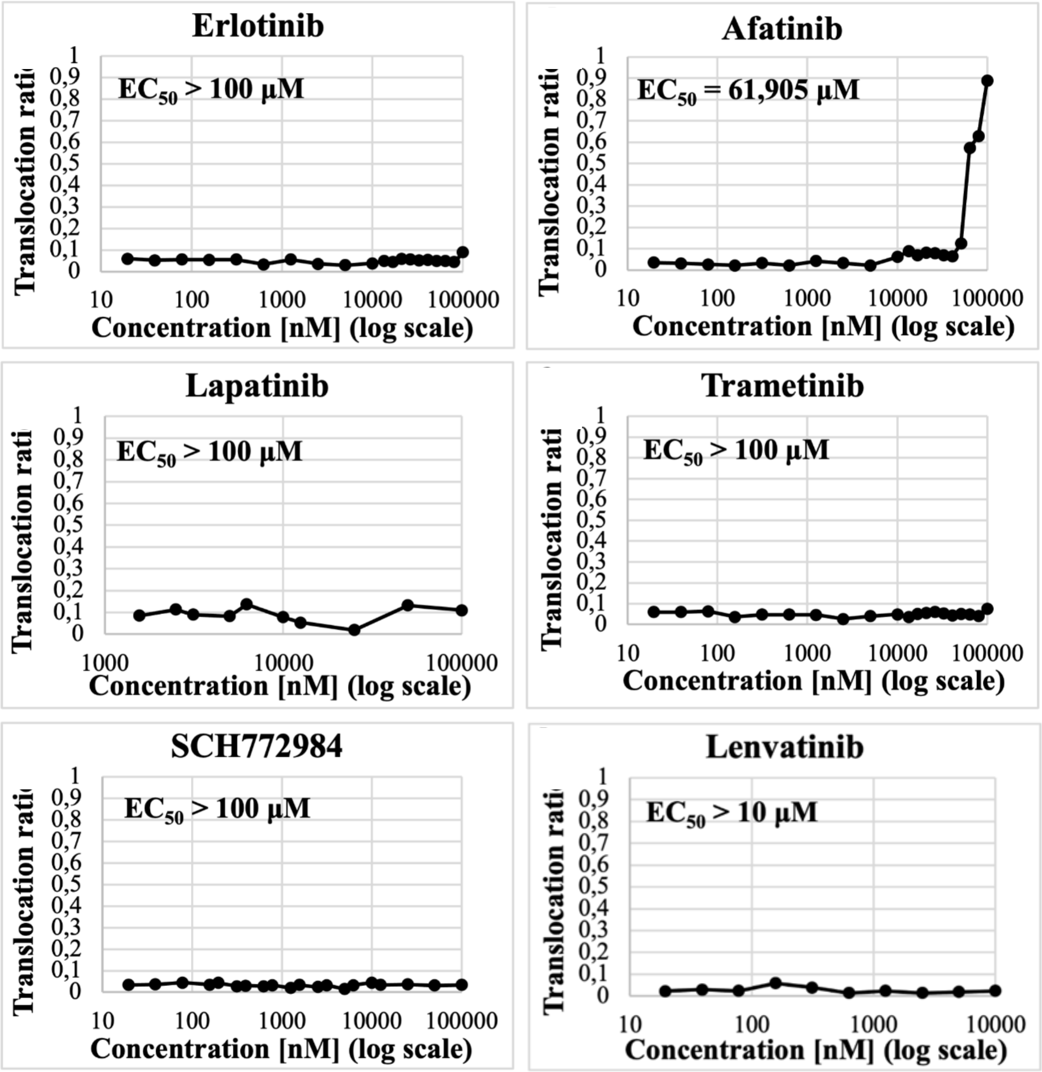

Supplementary Figure S1. Dose-response assay of RTKs and MEK/ERK inhibitors with U2foxRELOC cells. FOXO3 nuclear localization was quantified and represented as translocation ratio against each concentration in nM in logarithmic scale and then EC_{50} values were calculated. The compounds were tested in serial dilutions from 10mM to 10nM.

Supplementary Figure S2

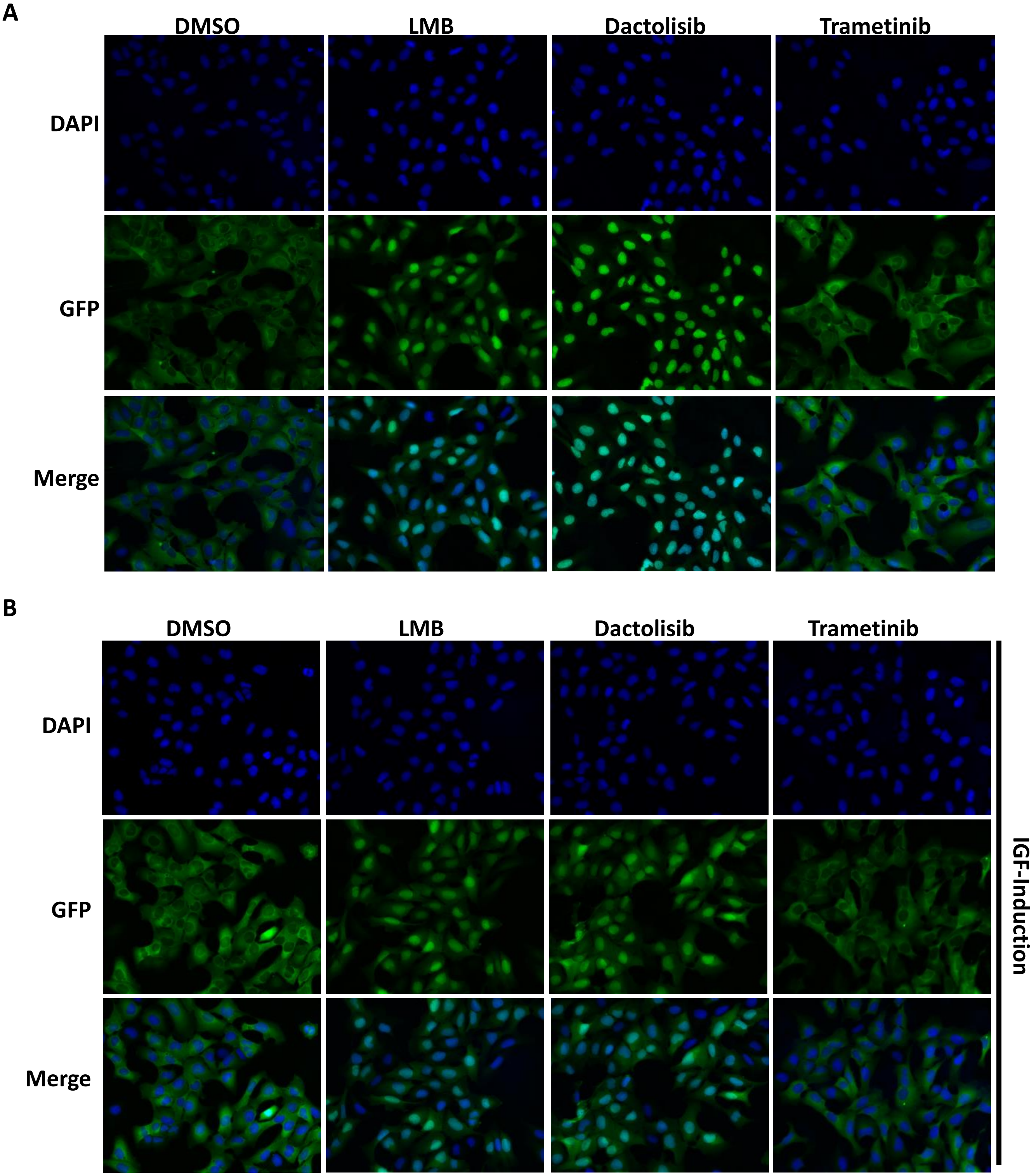

Supplementary Figure S2. FOXO translocation in U2foxRELOC under basal and EGF-induced conditions. **A)** U2foxRELOC in the absence or EGF treated with vehicle (DMSO), 20nM Leptomycin B (LMB), 500nM Dactolisib and 500nM Trametinib for 1 hour. Then cells were fixed and nuclear stained with DAPI. Representative images are shown at 20x magnification. **B)** FOXO translocation in U2foxRELOC in the presence of 50ng/ml EGF. Cells were treatment as in A).

Supplementary Figure S3

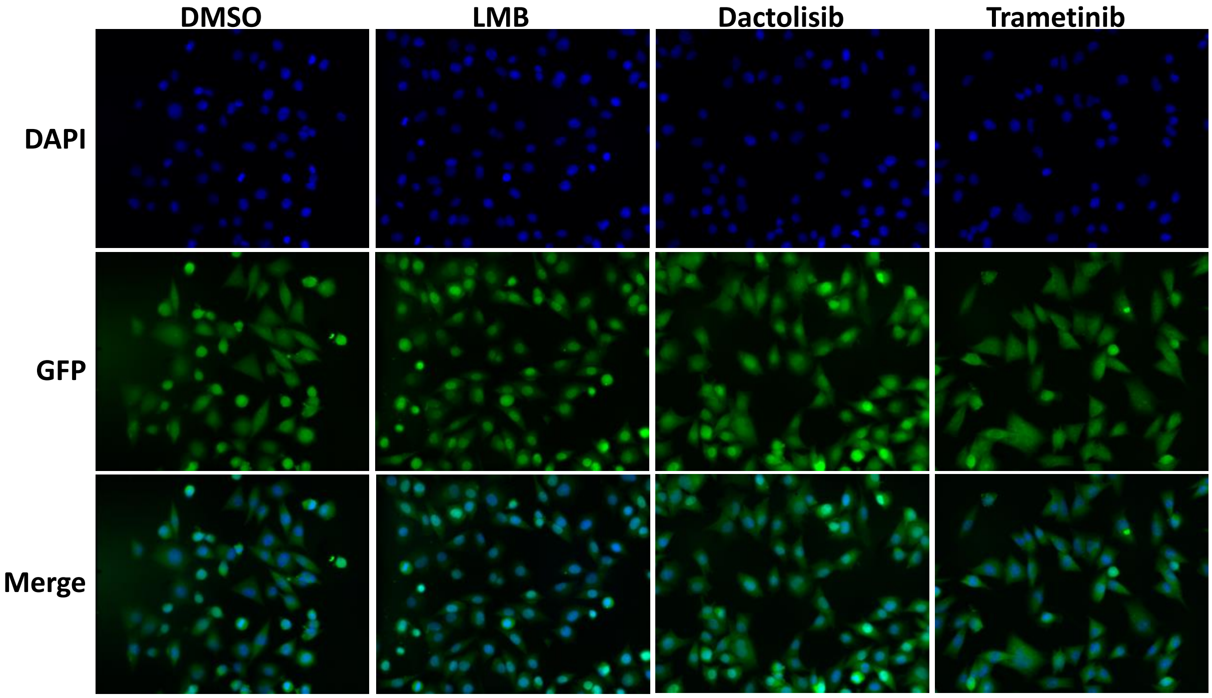

Supplementary Figure S3. FOXO translocation in UACC62 melanoma cells. UACC62 were treated with vehicle (DMSO), 20nM Leptomycin B (LMB), 500nM Dactolisib and 500nM Trametinib for 1 hour. Then cells were fixed and nuclear stained with DAPI. Representative images are shown at 20x magnification.

Supplementary Figure S4

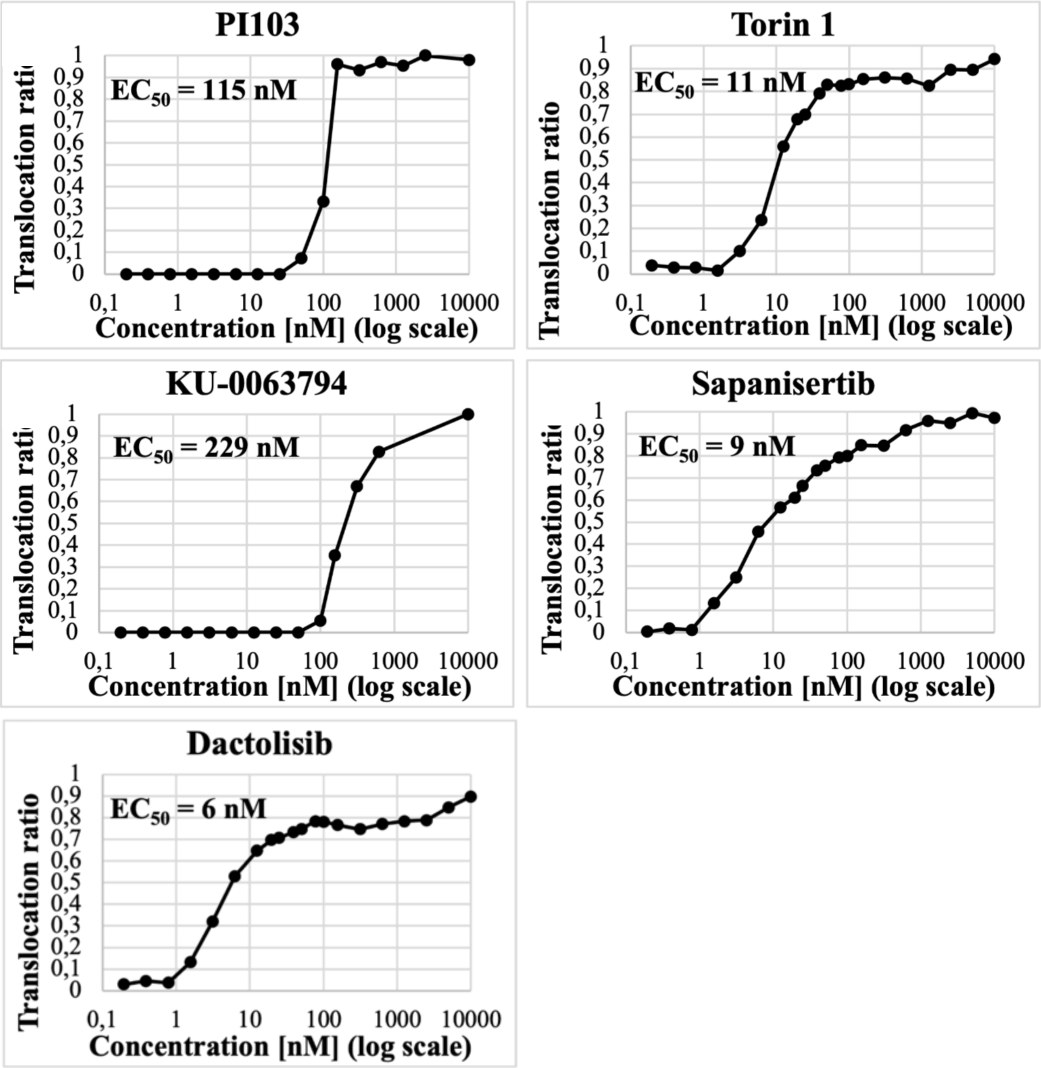

Supplementary Figure S4. EC_{50} concentration of PI3K and mTOR inhibitors. The compounds were tested in serial dilutions from 10mM to 10nM in U2foxRELOC cells. FOXO3 nuclear localization was quantified and represented as translocation ratio against each concentration in nM in logarithmic scale and then EC_{50} values were calculated.

Supplementary Figure S5

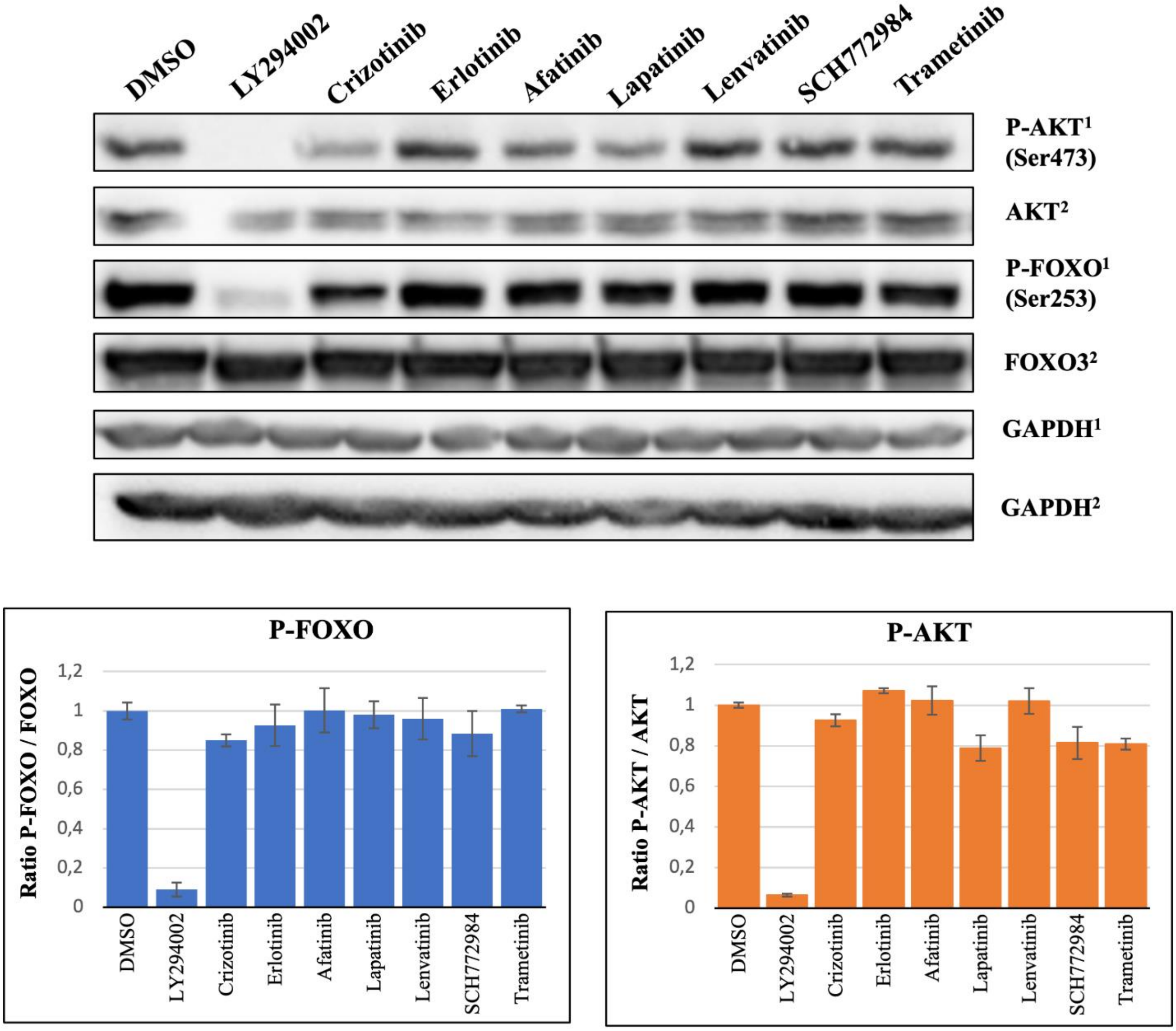

Supplementary Figure S5. Western Blot analysis of RTKs and MEK/ERK inhibitors. U2foxRELOC cells were cultured and treated for 1 hour with DMSO 1 % (negative control), LY294002 25 µM (positive control) or small molecule compounds (crizotinib, erlotinib, afatinib, lapatinib, Lenvatinib, SCH772984 and trametinib) at 1 µM concentration, and then were immunoblotted with specific antibodies against P-FOXO3 (Ser253) and total FOXO3, P-AKT (Ser473) and total AKT, and GAPDH as loading control. P-AKT and P-FOXO are labelled with [1] and should be compared with the loading control form the same membrane labelled as β-Actin[1], while β-Actin[2] represents the control for total AKT and FOXO3. Plots represent the quantification of P-FOXO3 and P-AKT normalized by the correspondent total protein and loading control and compared to DMSO negative control. Mean of triplicates and standard deviation are represented.
